# Loop-mediated isothermal amplification (LAMP)/Cas12a assay for detection of *Ralstonia solanacearum* in tomato

**DOI:** 10.3389/fbioe.2023.1188176

**Published:** 2023-05-22

**Authors:** Zhiyu Fan, Yuxia Mei, Jiawei Xing, Tian Chen, Di Hu, Hui Liu, Yingjun Li, Derui Liu, Zufeng Liu, Yunxiang Liang

**Affiliations:** ^1^ State Key Laboratory of Agricultural Microbiology, College of Life Science and Technology, Huazhong Agricultural University, Wuhan, China; ^2^ GNSS Research Center, Wuhan University, Wuhan, China; ^3^ Hubei Jiamachi Ecological Agriculture Co, Ltd, Yichang, China; ^4^ Hubei Yishizhuang Agricultural Technology Co, Ltd, Yichang, China

**Keywords:** CRISPR/Cas12a, LAMP, Ralstonia solanacearum, bacterial wilt (BW), detection

## Abstract

**Introduction:** Bacterial wilt (BW) caused by the aerobic, Gram-negative pathogenic species *Ralstonia solanacearum* (RS) is a major disease impacting commercial agriculture worldwide. Asian phylotype I of RS is the cause of tomato bacterial wilt, which has caused severe economic losses in southern China for many years. An urgent priority in control of bacterial wilt is development of rapid, sensitive, effective methods for detection of RS.

**Methods:** We describe here a novel RS detection assay based on combination of loop-mediated isothermal amplification (LAMP) and CRISPR/Cas12a. crRNA1, with high trans-cleavage activity targeting *hrpB* gene, was selected out of four candidate crRNAs. Two visual detection techniques, involving naked-eye observation of fluorescence and lateral flow strips, were tested and displayed high sensitivity and strong specificity.

**Results and Discussion:** The LAMP/Cas12a assay accurately detected RS phylotype Ⅰ in 14 test strains, and showed low detection limit (2.0 × 10^0^ copies). RS in tomato stem tissue and soil samples from two field sites with suspected BW infection was identified accurately, suggesting potential application of LAMP/Cas12a assay as point-of-care test (POCT). The overall detection process took less than 2 h and did not require professional lab equipment. Our findings, taken together, indicate that LAMP/Cas12a assay can be developed as an effective, inexpensive technique for field detection and monitoring of RS.

## 1 Introduction

Bacterial wilt (BW) is a major soil-borne bacterial plant disease caused by *Ralstonia* solanacearum (RS), an aerobic, non-spore-forming, Gram-negative species (phylum Pseudomonadota, class Betaproteobacteria) (Yabuuchi et al., 1995), one of the top ten bacterial plant pathogens worldwide (Mansfield et al., 2012). RS is widely distributed in tropical, subtropical, and temperate regions (Hayward, 1964), and is capable of infecting >400 plant species, including a wide range of commercial crops (Mansfield et al., 2012). RS is genetically diverse, as revealed by phylogenetic analysis; it is generally considered a species complex subdivided into four phylotypes (I, II, III, IV) (Fegan and Prior, 2005; Xue et al., 2009; Tan et al., 2011). Tomato bacterial wilt (TBW), a common plant disease in China, is mainly caused by Asian phylotype I, which is the predominant strain of the pathogen in Chinese bacterial wilt (Xue et al., 2011). In addition, this pathogen which classified in phylotype I can also infect a variety of crops such as potato (Liu et al., 2017), mulberry (Huang et al., 2017), and tobacco (Li et al., 2016), causing significant damage to agricultural production.

TBW is a severe problem in provinces south of the Yangtze River; its incidence ranges from 10%–80% depending on crop season (Jiang et al., 2017). There is no effective, environmentally friendly strategy for prevention or control of TBW. An urgent priority is development of a detection method having high efficiency, high sensitivity, and strong specificity. Such a method would ideally provide effective early warning in relation to agricultural production, and significantly reduce the impact of BW outbreaks and economic losses.

Many approaches have been used for detection of RS; these include selective media (Aley and Elphinstone, 1995), serological techniques (Panferov et al., 2016), and molecular biological testing (Chen et al., 2010). Tripheny tetrazolium chloride (TZC) medium was the first to be used for specific RS screening (Kelman, 1954), and was gradually replaced by modified selective medium South Africa (M-SMSA). Selective media allow determination of viable bacteria quantity and pathogenicity; however, this approach is time-consuming and not well suited for complex sample testing (Aley and Elphinstone, 1995). Numerous serological techniques for RS detection have been developed since the 1990s, including enzyme-linked immunosorbent assay (ELISA) (Caruso et al., 2002; Bellstedt, 2009), lateral flow immunoassay (LFIA) (Panferov et al., 2016; Ma et al., 2018; Razo et al., 2019), immunochromatographic test strip (ITS) assay (Tian et al., 2022), and time-resolved fluorescent immunochromatographic (TRFIC) assay (Fan et al., 2022). These techniques have higher sensitivity and stronger specificity relative to selective media; however, the process of establishing these methods is time-consuming, and has a potential of high false positive rate resulting from cross-reactions.

Nucleic acid detection has been used increasingly as molecular biology and PCR technology has progressed. Opina et al. (1997) identified RS using PCR technology by targeting a conserved sequence in the upstream region of LpxC gene. Huang et al. (2009) applied real-time PCR technology for quantitative detection of RS in soil. PCR has been used extensively in a variety of fields; however, its requirement for large, expensive thermal cyclers greatly limits its applicability in resource-limited settings and for point-of-care test (POCT) (Zhao et al., 2015). Isothermal amplification of nucleic acids is a promising recent alternative in which rapid, efficient amplification is achieved at constant temperature without thermocycling (Deng and Gao, 2015; Jia and Joanne, 2015; Becherer et al., 2020). The loop-mediated isothermal amplification (LAMP) method for rapid RS detection without requirement of expensive instruments was developed subsequently (Huang et al., 2017). However, elimination of non-specific amplification is a major obstacle to optimization of accurate LAMP assays (Liu et al., 2017; Meagher et al., 2018; Rolando et al., 2020; Varona et al., 2021).

Clustered Regularly Interspaced Short Palindromic Repeats (CRISPR) technology has been rapidly developed and widely used during the past 5 years for accurate genome editing and nucleic acid detection (Aman et al., 2020; van Dongen et al., 2020; Zhu et al., 2020). CRISPR/Cas12a systems have the potential to overcome the above-described problems in RS detection, and improve detection accuracy. CRISPR/Cas12a can bind to crispr RNA (crRNA) to form a Cas12a-crRNA complex, which then locates a short T-rich sequence (5′-TTTV-3′) protospacer adjacency motif (PAM) and recognizes a specific target DNA sequence to trigger trans-cleavage activity for cleavage of nonspecific single-stranded DNA (ssDNA) (Yamano et al., 2016; Swarts and Jinek, 2019). Pathogens carrying specific target genes can be identified by designing appropriate crRNA and incorporating an ssDNA probe in the detection system. This strategy has been applied with some success for detection of coronavirus (SARS-CoV-2) (Broughton et al., 2020; Joung et al., 2020), human papillomavirus (HPV) (Chen et al., 2018), and certain types of pathogenic bacteria (Yao et al., 2018; Qian et al., 2022). In order to increase detection sensitivity, CRISPR/Cas12a systems have been combined with nucleic acid amplification techniques, including PCR (Zhang et al., 2020; Mu et al., 2022), LAMP (Pang et al., 2020; Yang et al., 2022), recombinant polymerase amplification (RPA) (Luo et al., 2021), and recombinant enzyme-assisted amplification (RAA) (Bai et al., 2019; Qian et al., 2022). Combining LAMP with the CRISPR/Cas12a system has the potential to be tested under simple conditions with high sensitivity and feasibility. The false positivity risk of LAMP can be greatly reduced by CRISPR/Cas12a crRNA through conserved target sequence design (Pang et al., 2020). Thus, combination of LAMP with CRISPR/Cas12a system have potential for visible detection of RS, which is expected to meet the requirements of a POCT.

We report here establishment of a CRISPR/Cas12a platform integrated with LAMP for accurate detection of RS (phylotype I), and of two effective result reading methods: fluorescence and lateral flow strips (LFS). A highly conserved *hrpB* gene that encodes HrpB transcriptional regulon, which controls Type III secretion system (T3SS) and Type III effector (T3E) gene expression (Alfano and Collmer, 1997; Lindgren, 1997; Genin and Denny, 2012; Ma et al., 2018), was selected as the target DNA. The method was successfully applied for identification of RS in naturally infected tomato tissue and soil samples.

## 2 Materials and methods

### 2.1 Materials and reagents

Seven RS strains were cultured in NB medium (glucose 10.0 g/L, peptone 5.0 g/L, yeast extract 0.5 g/L, beef extract 3.0 g/L) for 24 h at 30°C, 180 rpm, and identified by phylotype-specific multiplex PCR on ITS sequences (Supplementary Figure S1) (Fegan and Prior, 2005). A closely related *Ralstonia* species [*Ralstonia pickettii* (Rp)], three common plant pathogens [*Rhizobium rhizogenes* (Rr), *Agrobacterium tumefaciens* (At), *Xanthomonas campestris* (Xc)], and three soil microorganisms (*Acinetobacter baumannii* (Ab), *Enterobacter hormaechei subsp* (Ehs), *Enterobacter cancerogenus* [Ec]) [details in Supplementary Table S1 (S: Supplementary material)] were incubated in LB medium (peptone 10.0 g/L, yeast extract 5.0 g/L, NaCl 10.0 g/L) for 24 h at 37°C, 180 rpm.

EnGen LbaCas12a (Cpf1) (cat #M0653T), dNTP mix (#N0447S), Bst2.0 DNA Polymerase (#M0275), Monarch RNA Cleanup Kit (#T2050S), DNase I (#M0303S), and HiScribe T7 High Yield RNA Synthesis Kit (#E2040S) were from New England Biolabs (Ipswich, MA, United States). AceQ universal SYBR qPCR Master Mix (#Q511-02) and 2 × Rapid Taq Master Mix (#P222-01) were from Vazyme Biotech (Nanjing). PrimeSTAR Max DNA Polymerase (#R045A), RNase-free water (#9012), and Recombinant RNase Inhibitor (#2313A) were from Takara (Tokyo). TIANamp Bacteria DNA kit (#DP302) and Plant Genomic DNA kit (#DP305) were from Tiangen Biotech (Beijing). GeneJET Plasmid Miniprep Kit (#K0503) and Finntip Filtered Pipet Tips (#94052000) were from Thermo Scientific (Waltham, MA, United States). E.Z.N.A. Soil DNA Kit (#D5625-01) was from Omega Bio-Tek (Norcross, GA, United States). HybriDetect—universal Lateral Flow Assay Kit (#MGHD-1) was from Milenia Biotec (Gießen, Germany). Mineral oil was from Beyotime Biotechnology (Shanghai). Chemical reagents were from Sinopharm Chemical Reagent Co. (Shanghai).

### 2.2 Preparation of genomic DNA and plasmid pUC57-*hrpB*

RS strains were streaked onto TZC agar (peptone 10.0 g/L, glucose 5.0 g/L, hydrolyzed casein 1.0 g/L, agar 18.0 g/L, tetrazolium chloride 0.5 g/L), and incubated for 48 h at 30°C. Single colonies with slimy, milky appearance and pink center were transferred to NB medium (glucose 10.0 g/L, peptone 5.0 g/L, yeast extract 0.5 g/L, beef extract 3.0 g/L) and incubated for 24 h at 30°C, 180 rpm. Other bacteria were cultured in LB medium (peptone 10.0 g/L, yeast extract 5.0 g/L, NaCl 10.0 g/L) for 24 h at 28°C, 180 rpm. Genomic DNA was extracted and purified using TIANamp Bacteria DNA kit as per manufacturer’s instructions.

A 215-bp fragment of RS *hrpB* gene was synthesized, linked to vector pUC57 to obtain recombinant plasmid pUC57-*hrpB* (Supplementary Figure S2), and sequenced by Tsingke Biotechnology Co. (Wuhan) pUC57-*hrpB* was used as positive control in subsequent experiments. Plasmid DNA was extracted using GeneJET Plasmid Miniprep Kit (Thermo Fisher). DNA concentrations were determined by measuring absorbance (NanoDrop One spectrophotometer, Thermo Fisher).

### 2.3 Cas12a crRNA design and synthesis

CrRNA sequence was designed online (www.rgenome.net) based on adjacent motif of PAM (TTTV) of *hrpB* gene. Four crRNAs with high scores (Supplementary Table S2) were selected as candidates. For crRNA preparation, DNA oligo (100 µM), T7-cr-oligo (containing T7 promoter, guide sequence, and crRNA scaffold sequence), and T7-top were synthesized by Tsingke Biotechnology. For double-stranded DNA (dsDNA) annealing, T7-cR-oligo (100 μM) and T7-top (100 μM) were denatured at 95°C for 5 min, kept at room temperature for 1 h, and annealed dsDNA was used as transcription template. CrRNA was synthesized from annealed dsDNA by 16 h incubation at 37°C using HiScribe T7 High Yield RNA Synthesis Kit. Transcriptional crRNA was purified using Monarch RNA Cleanup Kits (New England Biolabs; Beijing), analyzed by gel electrophoresis, and stored at −80°C.

### 2.4 Verification of Cas12a trans-cleavage activity

PCR amplification of full-length *hrpB* gene (825 bp) was performed in 50 μL reaction mixture using primer pair RShrpbF/RShrpbR (Supplementary Table S3) (10 μM, 2 μL), 25 μL PrimeSTAR HS (Premix), 100 ng DNA template, total volume 50 μL by addition of RNase-free water. Program: 98°C for 5 min; 35 cycles of 98°C for 10 s, 55°C for 10 s, 72°C for 50 s; 72°C for 5 min. The resulting PCR product were analyzed by 1% agarose gel electrophoresis.

To evaluate ability of a candidate crRNA to activate LbaCas12a trans-cleavage activity, we used an assay system containing FQ-reporter (5′FAM-TTATT-3′BHQ1), *hrpB* gene PCR product, LbaCas12a, and purified crRNA. FQ-reporter composed of FAM fluorophore and quencher was synthesized by Tsingke Biotechnology. A mixture system (30 μL) containing 1× NEBuffer 2.1, 750 nM FQ-reporter, 300 nM crRNA, 100 nM LbaCas12a, and 3 μL PCR product was incubated in CFX Connect Real-Time PCR Detection System (Bio-Rad; Irvine, CA, United States) for 1 h at 37°C. Resulting fluorescence was measured on a fluorescence plate reader (FAM channel), with plates read every 1 min. Cis-cleavage activity was performed in a reaction system (10 μL) containing 1× NEBuffer 2.1, 300 nM crRNA, and 100 nM LbaCas12a. PCR product (3 μL) was incubated in FlexCycler2 Thermal Cycler (Analytik Jena; Jena, Germany) for 1 h at 37°C, and cleavage products were analyzed by gel electrophoresis.

### 2.5 LAMP assay

LAMP primers were designed to target *hrpB* gene sequences obtained from GenBank (accession #AF295618.1), using Primer Explorer V.4 software program (Supplementary Table S3), and synthesized by Tsingke Biotechnology. LAMP was performed as per the protocol of (Huang et al., 2017) with minor modifications. Reaction mixture (25 μL) (1.6 μM FIP and BIP, 0.2 μM F3 and B3, 1.4 mM dNTPs, 0.1% Tween-20, 1.0 M betaine, 20 mM Tris-HCl (pH 8.8), 10 mM (NH4)_2_SO_4_, 50 mM KCl, 8 mM MgSO4, 8 U *Bst* 2.0 DNA Polymerase, 2 μL template DNA, covered with a layer of mineral oil) was incubated for 35 min at 65°C in a FlexCycler2 Thermal Cycler (Analytik Jena), and resulting product was analyzed by 2% agarose gel electrophoresis.

### 2.6 LAMP/Cas12a-fluorescence assay

To establish this assay, LAMP reaction as above was performed as the first step. Product (3 μL) was transferred via filtered pipette tips to a reaction system (1× NEBuffer 2.1, 300 nM crRNA, 100 nM LbaCas12a, 750 nM FQ-reporter), and incubated for 1 h at 37°C. Fluorescence was measured as in Section 2.4. As the next step, the overall reaction was optimized in terms of cost-efficiency, based on numerous test values of LbaCas12a concentration (10, 16, 25, 33, 66, 100, 133, 166 nM), crRNA concentration (25, 50, 100, 140, 180, 200, 233, 466, 700, 933 nM), FQ-reporter concentration (200, 350, 500, 650, 800, 950, 1100, 1250 nM), and LAMP reaction time (10, 15, 20, 25, 30, 35, 40, 45, 50 min). A negative control (addition of 1250 nM FQ-reporter; RNase-free water as template) was run to eliminate effects of background signals. Samples were visualized by UV illumination. Experiments were performed in triplicate.

Sensitivity of the optimized LAMP/Cas12a-fluorescence assay was confirmed using plasmid pUC57-*hrpB* (10-fold serial dilution from 2 × 10^10^ to 2 × 10^-2^ copies) as template, in triplicate. Specificity of the assay was confirmed using genomic DNA (gDNA) of the strains and species described in Section 2.1 (first paragraph) and Supplementary Table S1.

Sensitivity was also evaluated using qPCR with F3/B3 primer of LAMP; program: 98°C for 5 min; 40 cycles of 98°C for 10 s, 55°C for 10 s, 72°C for 50 s; 72°C for 5 min; read plate once at end of each cycle. qPCR was run on CFX Connect Real-Time PCR Detection System, and sensitivity was determined based on cycle threshold (Ct) values at various template concentrations.

### 2.7 LAMP/Cas12a-lateral flow assay (LFA)

Variability of LAMP/Cas12a in application domain was amplified using LFA system, whereby positive band signals are observed at specific locations when antigen-antibody affinity complex passes through a lateral flow strip. Specific ssDNA was designed for use as antigen in biological affinity testing. LAMP/Cas12a-LFA was performed under optimized LAMP/Cas12a-fluorescence assay conditions as described above. FB-reporter (5′6-FAM-TTTTTATTTTT-3'biotin) was used instead of FQ-reporter, and its concentration was optimized (Supplementary Figure S3). Reaction was performed in a tube for 40 min at 37°C, for 40 min, 80 μL 5% polyethylene glycol (PEG) buffer was added, and the strip was placed in the tube and incubated for 3 min at room temperature. Visible color developed in the test-line (T-line) and control band within 5 min. Sensitivity and specificity of LAMP/Cas12a-LFA were similar to those of LAMP/Cas12a-fluorescence assay. The strip images were converted to 8-bit grayscale using software ImageJ (https://imagej.net/software/imagej/) and determined the gray value of T-line intensity.

### 2.8 LAMP/Cas12a assays of naturally infected samples

Naturally infected samples (stem tissues, soils) were collected from tomato plants and fields with suspected bacterial wilt in Changyang, Hubei Province, and Baise, Guangxi Province. Soil sample gDNA was extracted using E.Z.N.A. Soil DNA Kit as per manufacturer’s instructions. For stem tissue sample gDNA extraction, 1-cm piece of sample was cut with sterile scissors, washed with sterile water, sterilized with 70% ethanol, placed in a Petri dish with 10 mL sterile water, chopped, and suspended for 30 min gDNAs of stem tissue and soil samples were used as templates for LAMP/Cas12a-fluorescence assay and LAMP/Cas12a-LFA, and PCR amplification was performed simultaneously. PCR system (50 μL) consisted of primer pair 759/760 (Supplementary Table S3) (10 μM, each 2 μL), PrimeSTAR HS (Premix) (25 μL), DNA template (100 ng), and double-distilled water; program: 98°C, 5 min; 35 cycles of 98°C for 10 s, 55°C for 10 s, 72°C for 50 s; 72°C for 5 min.

### 2.9 Statistical analysis

Experimental data were expressed as mean ± SD, and differences between means were analyzed by one-way ANOVA using GraphPad Prism V. 8.0 software program (San Diego, CA, United States). Differences with *p* < 0.05 or <0.01 were considered significant or highly significant, respectively.

## 3 Results

### 3.1 Determination of RS-specific crRNA

**FIGURE 1:**
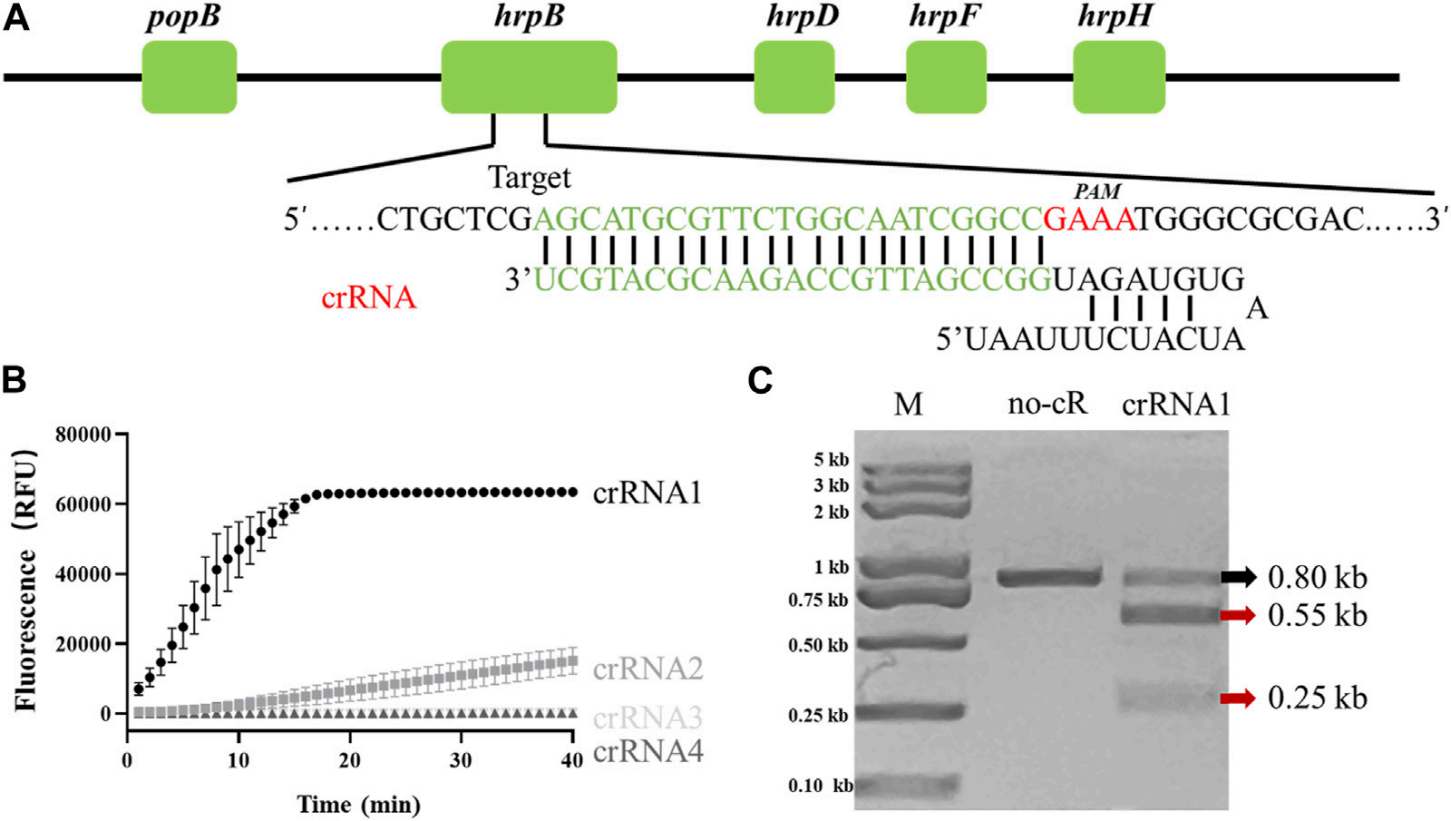
Determination of *R. solanacearum* (RS)-specific crRNA targeting *hrpB* gene. **(A)** Target site and crRNA sequence of RS *hrpB* gene (schematic). **(B)** Real-time fluorescence curves of PCR/Cas12a-FQ-reporter reactions with differing crRNAs. **(C)** *In vitro* dsDNA substrate cleavage assay. No-cR: no crRNA1 in reaction mixture (negative control). CrRNA1: crRNA1 was added to reaction mixture. Red arrows: expected cleavage products.

Fluorescence intensity of LAMP/Cas12a reaction system is presumably affected by differing crRNAs. The target gene locus recognized by crRNAs is a PAM sequence (TTTV) which is not present in all target genes. Identification of a gene suitable for crRNA design and evaluation was necessary. Various crRNAs were incubated with Cas12a, synthetic *hrpB* gene PCR product, and FQ-reporter for evaluation of crRNA efficiencies. Sites of candidate target genes are shown in Figure 1A.

Among four candidates, only crRNA1 and crRNA2 showed notable fluorescence. Fluorescence endpoint intensity was higher and repeatability better for crRNA1 than for crRNA2 (Figure 1B). To assess ability of crRNA1 to activate cis-cleavage, a 0.8-kb dsDNA substrate containing PAM sites was incubated with LbaCas12a and crRNA1. The products included two new bands (0.55-kb and 0.25-kb) (Figure 1C), whereas control reaction mixture (no crRNA1 addition) gave only 0.8-kb band, indicating that the substrate was cleaved at crRNA1 target position. Based on these results, we selected crRNA1 targeting *hrpB* gene for RS Phylotype I detection (Figure 1A).

### 3.2 Optimization of LAMP/Cas12a-fluorescence assay

**FIGURE 2:**
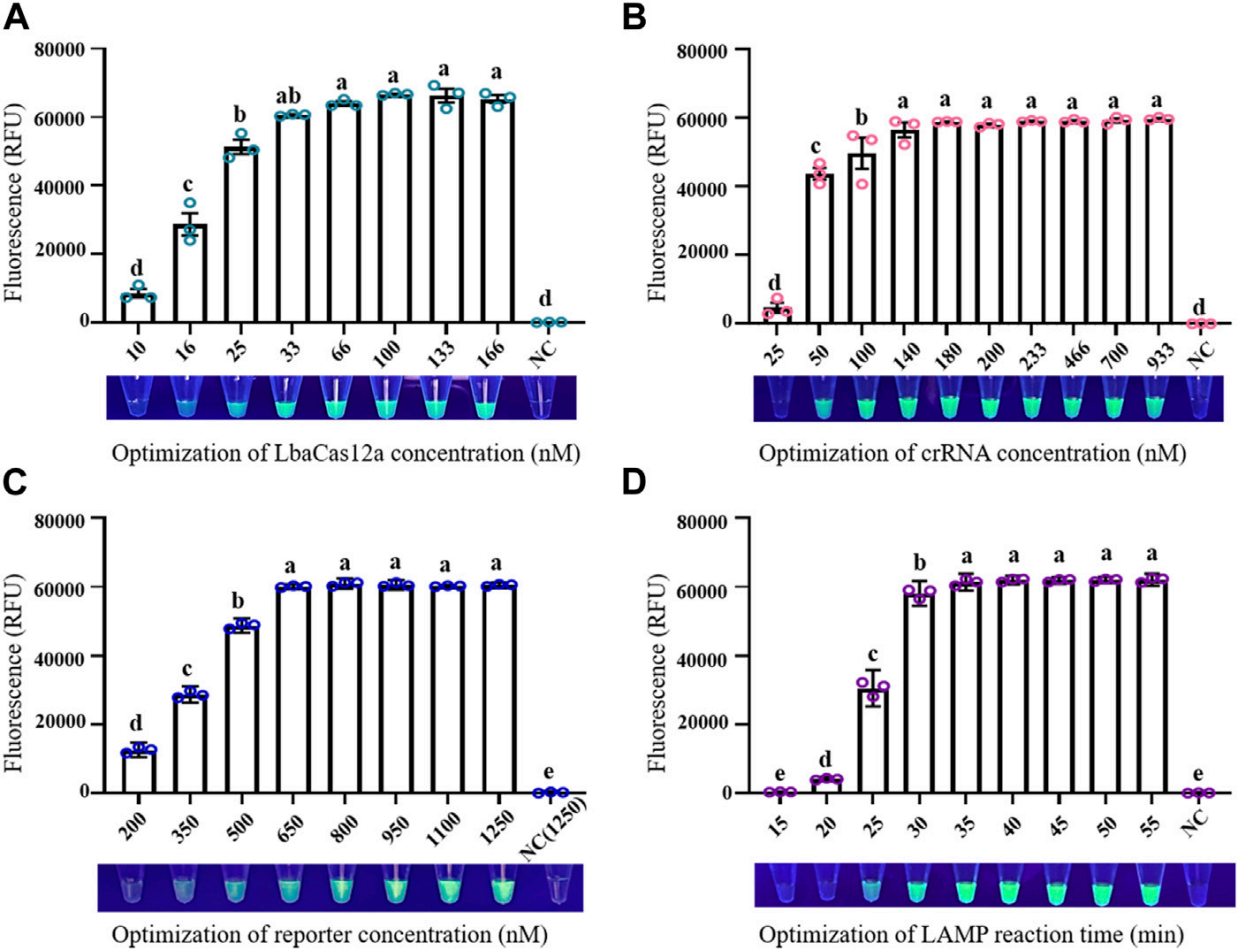
Optimization of factors in LAMP/Cas12a-fluorescence assay. **(A)** LbaCas12a concentration. **(B)** CrRNA concentration. **(C)** FQ-reporter concentration. **(D)** LAMP reaction time. NC: negative control (RNase-free water as template). Data shown are mean ± SD (*n* = 3). Differing letters above bars indicate significant (*p* < 0.05) differences by Tukey’s multiple comparison test.

Optimization of conditions was necessary for establishment of a reliable LAMP/Cas12a-fluorescence detection system. At LbaCas12a concentration 10 nM, the reaction system did not generate sufficient fluorescence to be observed under UV, and fluorescence value did not differ significantly from that of negative control (NC) (Figure 2A). Increase of LbaCas12a concentration in a certain range resulted in strong enhancement of overall fluorescence intensities. Fluorescence values began to plateau when LbaCas12a concentration reached 33 nM, and did not differ significantly in the 33–166 nM range. 33 nM was therefore selected as optimal LbaCas12a concentration, considering detection costs and final effects. Fluorescence intensities for crRNA concentrations ranging from 25 to 933 nM are shown in Figure 2B crRNA concentration 25 nM was clearly inadequate for activation of ssDNA cleavage by Cas12a, whereas fluorescence intensity and stability were notably better at concentration 50 nM. At crRNA concentrations ranging from 140–933 nM, reaction systems did not differ notably. 180 nM was selected as optimal crRNA concentration, considering detection accuracy and stability. Results for optimization of FQ-reporter concentration (tested range 200–1250 nM) are shown in Figure 2C Fluorescence value increased rapidly for concentrations 200–640 nM, and reached a plateau at 800 nM, which was selected as optimal FQ-reporter concentration. Results for optimization of LAMP reaction time in pre-amplification stage are shown in Figure 2D Fluorescence values increased rapidly for reaction times 5–30 min, and reached a plateau at 35 min, which was selected as optimal LAMP reaction time.

### 3.3 Sensitivity and specificity of LAMP/Cas12a-fluorescence assay

**FIGURE 3:**
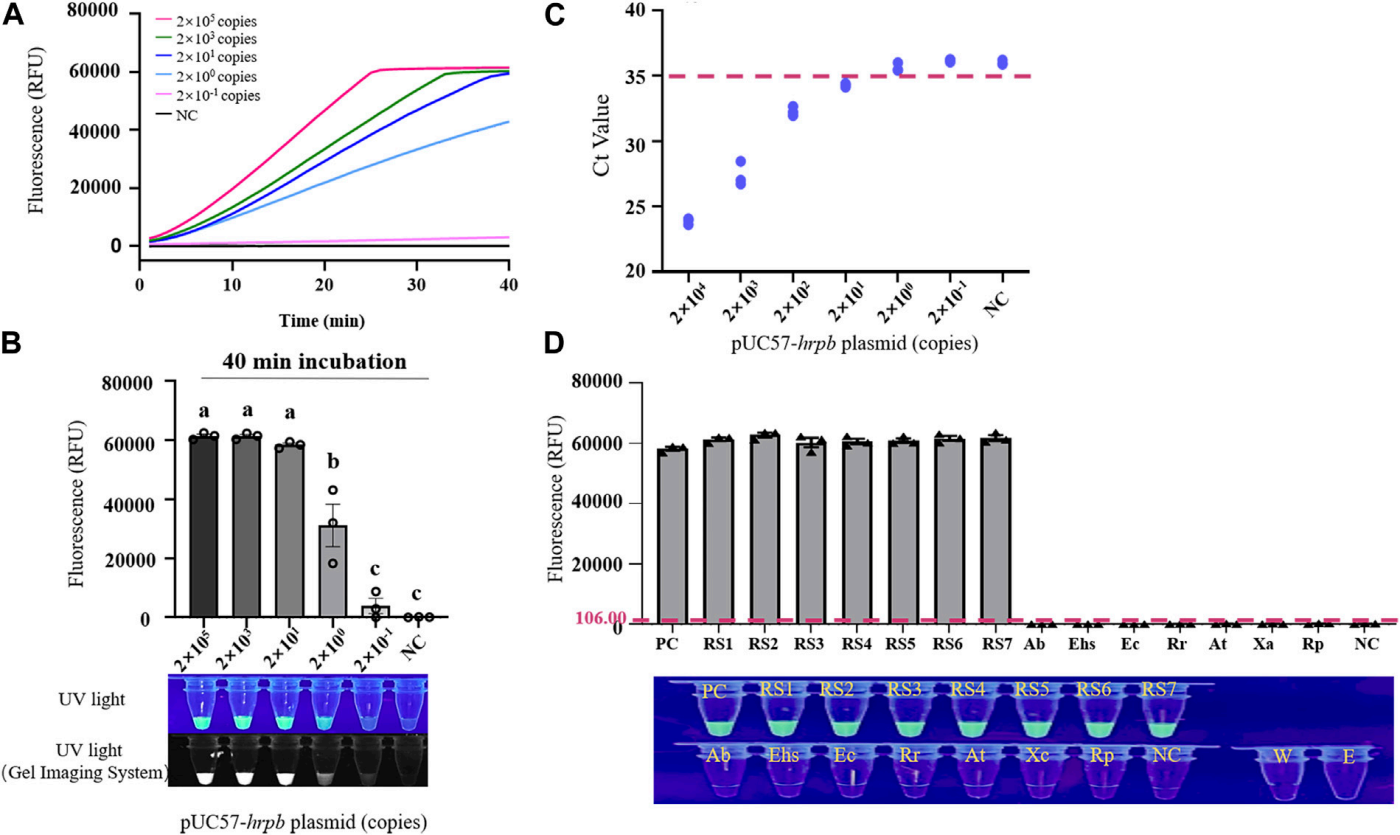
Sensitivity and specificity of LAMP/Cas12a-fluorescence assay. **(A)** Real-time LAMP/Cas12a-fluorescence detection of 10-fold serial dilution of pUC57-*hrpB*. **(B)** Endpoint of LAMP/Cas12a-fluorescence assay in above experiment. **(C)** Sensitivity of *hrpB* gene detection by RT-qPCR. Horizontal dashed line: threshold for determination of Ct value. **(D)** Detection of various strains by LAMP/Cas12a-fluorescence assay, with pUC57-*hrpB* as positive control template, and DEPC water as negative control (NC) template. Horizontal dashed line: fluorescence cutoff defined by average NC intensity plus three times SD. RS1-7: *Ralstonia solanacearum* phylotype Ⅰ; Ab: *Acinetobacter baumannii*; Ehs: *Enterobacter hormaechei subsp*; Ec: *Enterobacter cancerogenus*; Rr: *Rhizobium rhizogenes*; At: *Agrobacterium tumefaciens*; Xc: *Xanthomonas campestris*; Rp: *Ralstonia pickettii*; W: RNase-free water; E: empty tube. Statistical notations as in Figure 2.

Recombinant plasmid pUC57-*HrpB* (10-fold serial dilution to 2 × 10^-1^ copies/μL) was used as template for evaluation of sensitivity of LAMP/Cas12a-fluorescence assay for RS *hrpB* gene detection. Dynamic curve of LAMP/Cas12a-fluorescence (Figure 3A), and endpoint fluorescence intensities (Figure 3B), were analyzed. As pUC57-*HrpB* copy number declined from 2×10^5^ to 2 × 10^-1^, fluorescence intensity decreased gradually. Its value approached negative control value at copy number 2 × 10^-1^, and no fluorescence was observed under UV. Detection limit of LAMP/Cas12a-fluorescence was 2×10^0^ copies. Ct values > 35 from RT-qPCR are generally considered negative (Ke et al., 2022). RT-qPCR of RS *hrpB* gene showed detection limit 2×10^1^ copies; *i.e.*, sensitivity an order of magnitude higher than that of LAMP/Cas12a-fluorescence (Figure 3C). These findings indicate very high sensitivity of LAMP/Cas12a-fluorescence assay.

Specificity of LAMP/Cas12a-fluorescence assay was evaluated using seven phylotype I RS strains, with strains and species described in Section 2.1 and Supplementary Table S1 as negative samples. Fluorescence intensity values and results under UV (Figure 3D) revealed precise detection of RS phylotype I gDNA, whereas negative samples showed no fluorescence reaction. Thus, LAMP/Cas12a-fluorescence assay had strong specificity.

### 3.4 Optimization and evaluation of LAMP/Cas12a-LFA

For LAMP/Cas12a-LFA, FQ-reporter was replaced by FB-reporter, and its concentration was optimized (Supplementary Figure S3). FB-reporter concentration was strongly correlated with intensity of T-line band in negative control results, and optimal FB-reporter concentration was defined as 250 nM. Replacement of MGCB chase buffer (as recommended in HybriDetect—universal LFA Kit) by H_2_O+ 5% PEG 4000 significantly reduced false positive rate in negative control.

**FIGURE 4:**
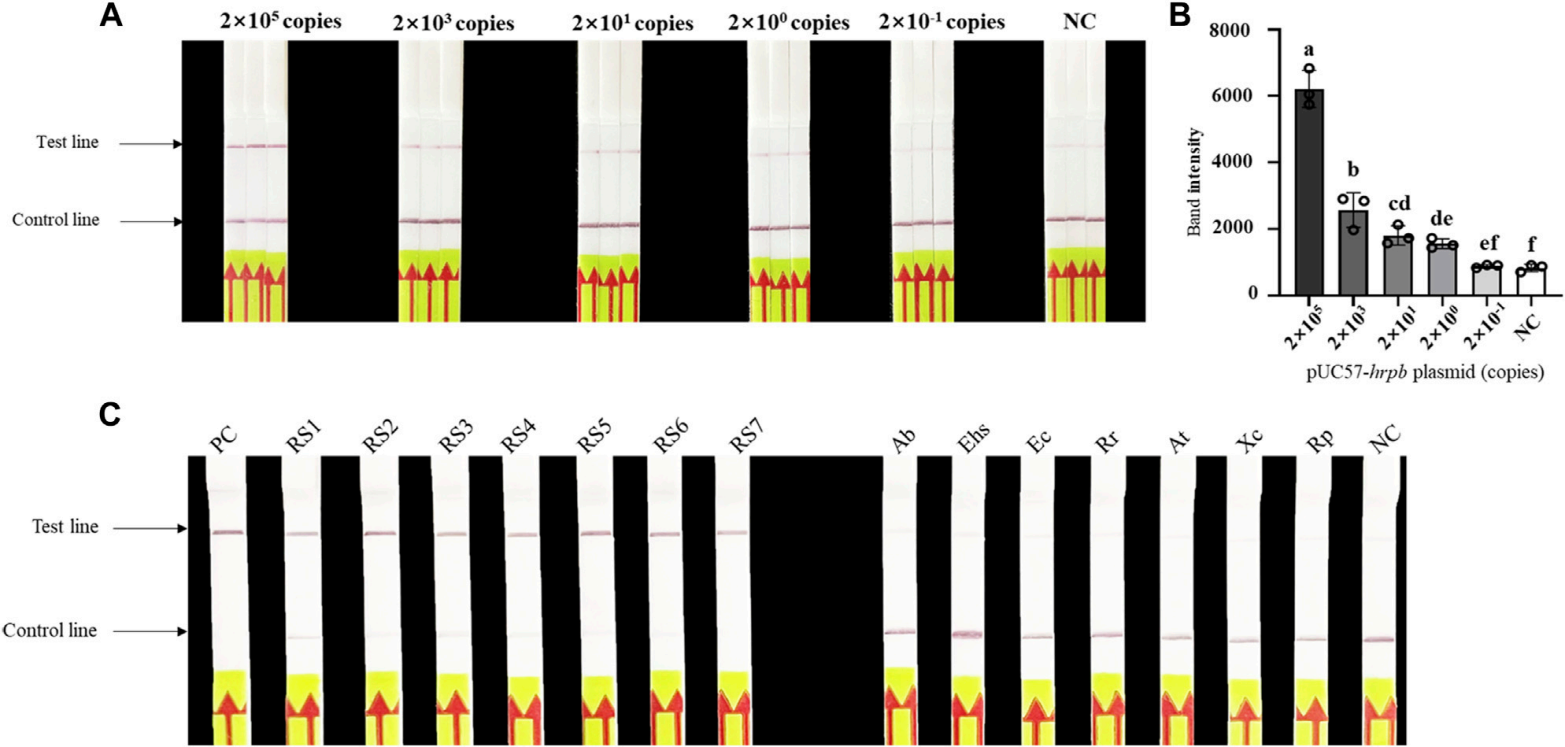
Sensitivity and specificity of LAMP/Cas12a-LFA. **(A)** Sensitivity analysis of LAMP/Cas12a-LFA. **(B)** Gray value analysis of results in **(A)** based on T-line band intensity. **(C)** Specificity analysis of LAMP/Cas12a-LFA using 14 test strains (abbreviations as in Figure 3).

Evaluation of sensitivity and specificity of LAMP/Cas12a-LFA (Figures 4A,B) indicated detection limit 2×10^0^ copies, consistent with results for LAMP/Cas12a-fluorescence assay. Seven RS phylotype I strains out of 14 test strains were accurately identified by LAMP/Cas12a-LFA (Figure 4C), again consistent with results for LAMP/Cas12a-fluorescence assay. Thus, LAMP/Cas12a-LFA also had strong specificity.

### 3.5 Detection of natural samples by LAMP/Cas12a assays

**FIGURE 5:**
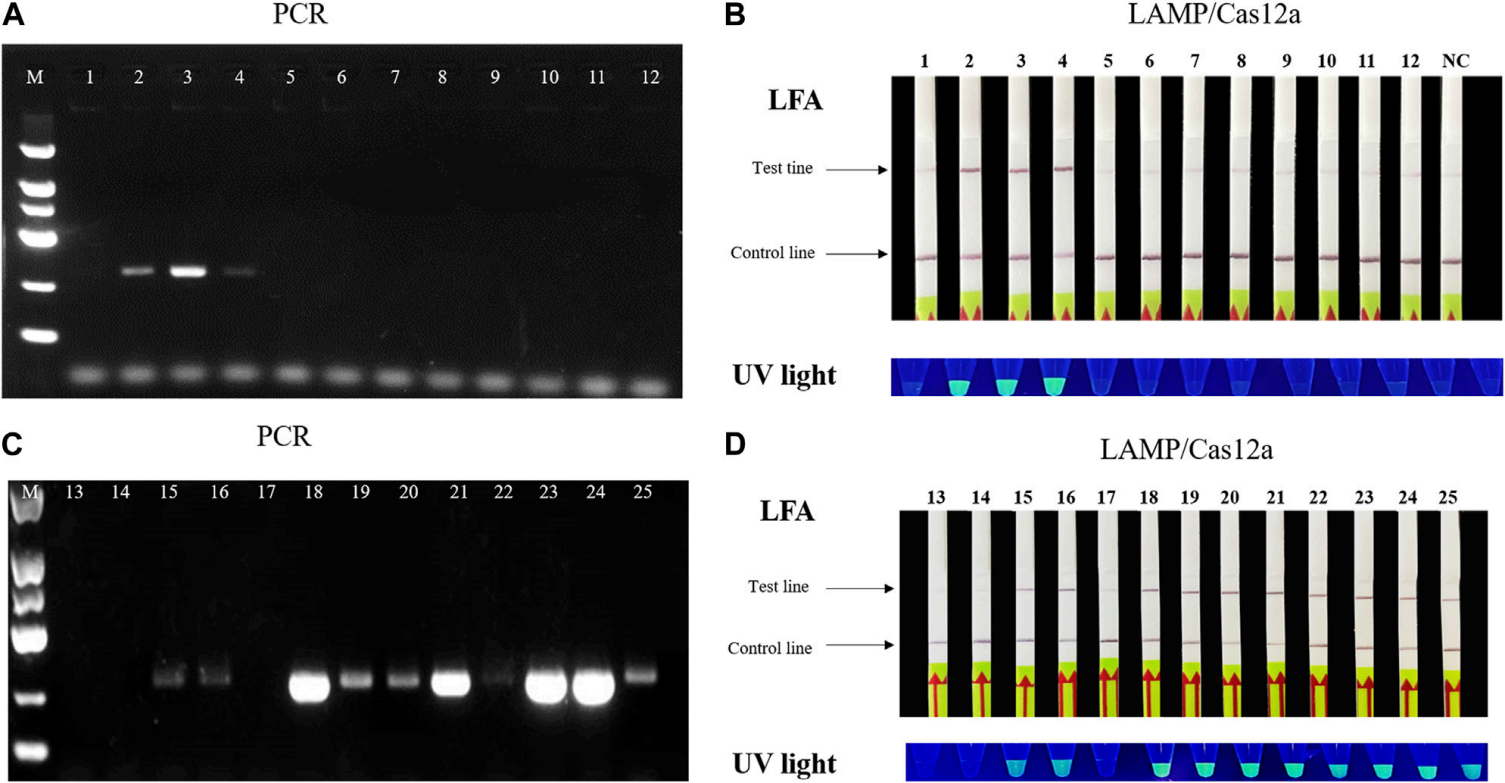
Application of LAMP/Cas12a-LFA to tomato stem tissue and soil samples. **(A)** PCR detection of soil samples (1–12). **(B)** LAMP/Cas12a-LFA of soil samples. **(C)** PCR detection of tomato tissue samples (13–25). **(D)** LAMP/Cas12a-LFA of tomato tissue samples. NC as in Figure 2.

Applicability of the two LAMP/Cas12a assay methods for natural plant tissue and soil samples was investigated using samples collected in Changyang and Baise (see Section 2.8). The assays indicated RS infection of soil samples No. 2-4, consistent with PCR detection results (Figures 5A,B). Furthermore, we detected RS infected plant samples with No. 15, 16 and 18–25, and the results were also consistent with that of PCR. Our findings demonstrate that the LAMP/Cas12a assays can specifically detect bacterial wilt disease even when the bacterial content in each plant sample varies (Figures 5C,D). Thus, the LAMP/Cas12a assays provided rapid, accurate, visual identification of RS in natural samples.

## 4 Discussion

Because BW occurs in forests and fields, development of a POCT for rapid field detection of RS is highly desirable. In generally, qPCR and next-generation sequencing (NGS) are the most commonly used methods for detection of many pathogenic microorganisms, including SARS-CoV-2 (Safiabadi et al., 2021), *Taenia solium* (pork tapeworm) (Wilson et al., 2018), and RS (Stulberg et al., 2016). The high sensitivity and excellent accuracy of qPCR make it the first choice in the event of any epidemic outbreak. However, qPCR requires expensive instruments which occupy a large amount of space, and the complex operating system must be performed by trained staff. qPCR also requires reading the CT value through a fluorescence quantometer in the process of experimental result reading. Due to the aforementioned drawbacks, the qPCR method is limited in meeting the rapid field detection of RS.

Various isothermal amplification techniques (e.g., RPA, LAMP) for nucleic acid detection have been developed (Zhao et al., 2015) and have greater prospects in the field of POCT. LAMP has a higher amplification efficiency and sensitivity than PCR, and the equipment used is portable, space-saving, and easy to operate. However, there are also some major shortcomings. Each reaction involves four pairs of primers, and dimers resulting from these primers lead to false positive errors (Schneider et al., 2019; Rolando et al., 2020). Traditional LAMP-based visual detection requires opening the lid and adding nucleic acid chromogens (Notomi et al., 2015), thus allowing environmental aerosol pollution (Zhang et al., 2014).

These problems can potentially be overcome by using a CRISPR/Cas12a system. LAMP primer binding target sequence triggers the first specific recognition event, and Cas12a protein plays an essential role in specificity enhancement. Cas12a cleavage activity requires mutual anchoring of ribonucleoprotein (RNP) complex and target sequence. Specific recognition of target sequence by Cas12a can eliminate false positives (Wang et al., 2021). The method described here essentially eliminates environmental aerosol pollution by i) covering the LAMP reaction system with mineral oil, and ii) transferring the end product to Cas12a reaction system with filtered pipette tips. We also used a nucleic acid cleaning kit to eliminate potential leaking of nucleic acid amplicons to the environment, and periodically ran PCR with sterile water as template during the experimental period to monitor potential aerosol contamination. Previous studies have applied LAMP assay and qPCR detection for RS identification (Kubota et al., 2008; Lenarčič et al., 2014; Li et al., 2021); however, the CRISPR/Cas12a assay described here has higher sensitivity. CRISPR/Cas12a cleavage of FQ-reporters can trigger second-round signal amplification of LAMP amplicons (Wang et al., 2021). LAMP in combination with CRISPR/Cas12a system has been used for single nucleotide polymorphism (SNP) detection, indicating the high accuracy and stability of this approach, and its potential utilization in a wide range of applications (Gootenberg et al., 2017). Our research has revealed that LAMP combined with CRISPR/Cas12a system for detecting RS exhibits greater detection sensitivity than qPCR.

**FIGURE 6:**
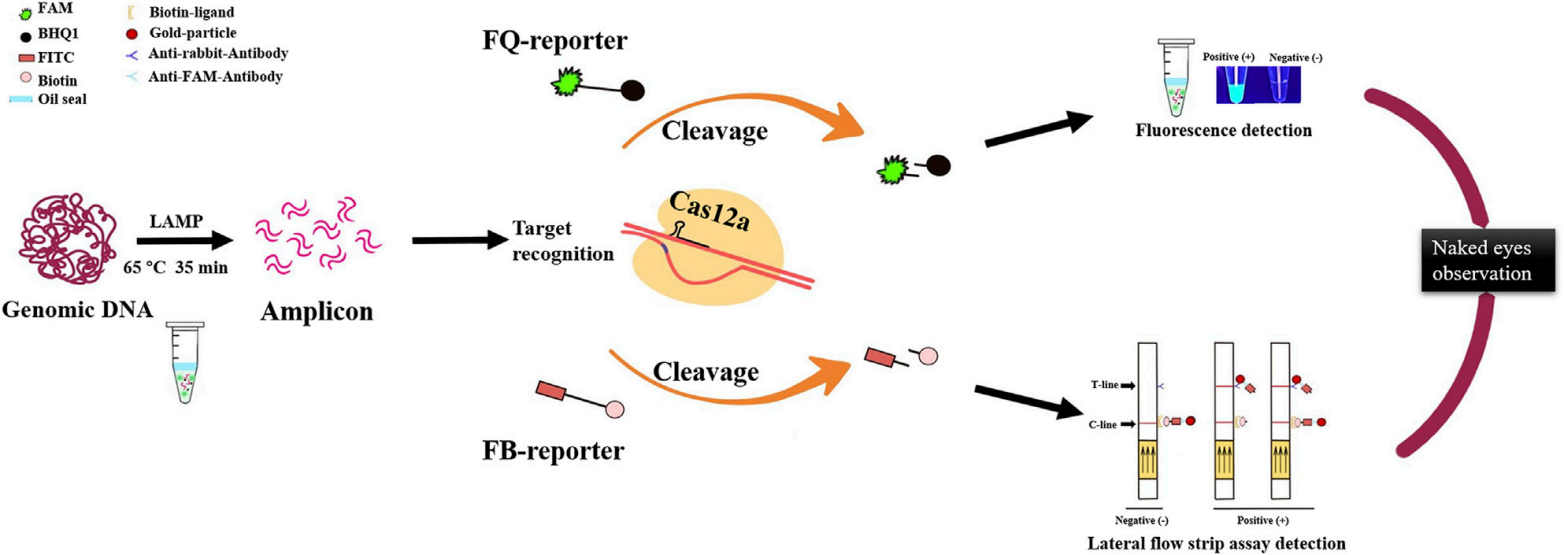
Optimized LAMP/Cas12a assay detection process for *R. solanacearum* with two visual methods for reading results: naked-eye observation of fluorescence and lateral flow strips.

In a word, the LAMP/Cas12a-based nucleic acid detection method described here is an accurate, efficient, sensitive POCT for rapid filed detection of RS. There are three stages in the detection process: LAMP, Cas12a cleavage reaction, and interpretation of results (Figure 6). Sensitivity and specificity of the method are ensured by reasonable design of LAMP primers and crRNA (Chen et al., 2022; Lu et al., 2022).

A critical issue in establishment of CRISPR/Cas12a assay is design of crRNA, which requires identification of a highly conserved target gene. Many genes were screened as nucleic acid detection targets in previous studies; these include *fliC* (which encodes flagellar subunit protein) (Schönfeld et al., 2003), *cytc1* (Kang et al., 2007), *hrp* (Singh et al., 2014), *egl* (Lenarčič et al., 2014), and *LpxC* (Okiro et al., 2019). The selected target gene in the present study was *hrpB*, which encodes regulatory protein HrpB in the type 3 secretion system (T3SS) of RS. We downloaded from National Center for Biotechnology Information (NCBI) database the *hrpB* gene sequences of all current reference sequevars of RS phylotype I, for multiple comparisons with corresponding gene sequences of other common plant pathogens. A 215-bp nucleotide fragment with PAM sequence (TTTV), having good conserved characteristics in RS phylotype I and only 20%–30% homology with other plant pathogens, was selected for crRNA design (Supplementary Figure S4). LAMP primers and crRNA sequences cannot be complementary and overlapping (Joung et al., 2020). Design of effective LAMP primer sets and crRNA sequences under these constraints is difficult—a major factor currently limiting Cas12a nucleic acid detection technology. An important goal in future research is detection of Cas12a without PAM sequence (Zhong et al., 2022).

Each phylotype in the RS species complex is associated with different hosts and pathogenicity (Prior et al., 2016; Paudel et al., 2020). Summarizing the detection methods for all pathogenic strains of RS species is challenging due to their severe differentiation and widely global distribution (Fegan and Prior, 2005; Genin and Denny, 2012). This study focuses on the detection of phylotype I in RS. Phylotype I originated in Asia is the dominant pathogenic strain in China, and cause severe TBW and even a significant threat to agricultural production. Rapid detection of Phylotype I meet the task of preventing and controlling BW in China. While, we believe that this method can also be applied to other phylotypes with minor modifications of design of crRNA and LAMP primers due to different sequences of the target gene (*hrpb*) in different phylotypes.

Some other potential limitations of LAMP/Cas12a assay also remain to be overcome in future studies. When RNase-free water was used as template for LFA detection, T-line for the negative control showed a faint band (Bai et al., 2019; Luo et al., 2021; Qian et al., 2022). For application of CRISPR/Cas12a-LFA, reporter concentration needs to be carefully optimized. Reporter concentrations that are too high or too low result in increased T-line background in negative control (Myhrvold et al., 2018). Here, we optimized reporter concentration, making the negative control band as small as possible. Replacement of MGCB chase buffer by 5% PEG increased the combined time of mobile phase and fixed phase, and minimized false positive signal value in negative control.

In view of unique characteristics of BW caused by RS, detection of RS in natural environment samples should be emphasized, and RS detection techniques should be focused on practical application. Here, we successfully identified RS in soil and tomato stem tissue samples with readable results within 2 h, indicating feasibility and high accuracy of LAMP/Cas12a assays. In this study, natural samples were collected from two areas in China where bacterial wilt disease is prevalent. The detection results using LAMP/Cas12a assays were verified by conventional PCR. Both results from our methods and PCR indicate that the samples without a signal were not infected. Thus, these samples could be sufficient to demonstrate the feasibility of this method in practical applications (Lei et al., 2022; Xu et al., 2022). Further study by collecting and detecting more samples from additional BW outbreak areas is undergoing. In a word, LAMP/Cas12a assays have strong potential application as POCT.

## 5 Conclusion

We developed a simple, rapid, accurate assay based on LAMP/Cas12a system for detection of *R. solanacearum* (RS), the pathogen causing bacterial wilt (BW), a major soil-borne plant disease. Following optimization of several key parameters, RS phylotype I was detected by LAMP/Cas12a assay within 2 h. Two visual methods (fluorescence and lateral flow strips) for reading results were established. LAMP/Cas12a assays showed higher sensitivity and stronger specificity in comparison with PCR detection, and their feasibility was verified based on detection of RS in naturally infected tomato stem tissue and soil samples. Our findings indicate strong potential of LAMP/Cas12a assays for development as commercial detection agents in agricultural production.

## Data Availability

The original contributions presented in the study are included in the article/Supplementary Material, further inquiries can be directed to the corresponding authors.
