# Supplementary material for: Loop-mediated isothermal amplification (LAMP)/Cas12a assay for detection of Ralstonia solanacearum in tomato
Source: Front Bioeng Biotechnol. 2023 May 22;11:1188176. doi: 10.3389/fbioe.2023.1188176 (PMC10239818; doi:10.3389/fbioe.2023.1188176)

Supplementary Material

**Loop-mediated isothermal amplification (LAMP)/Cas12a assay for detection of *Ralstonia solanacearum* in tomato**

**Zhiyu Fan^1^, Yuxia Mei^1*^, Jiawei Xing^1^, Tian Chen^1^, Di Hu^1^, Hui Liu^2*^, Yingjun Li^1^, Derui Liu^3,4^, Zufeng Liu^3,4^, Yunxiang Liang^1^**

*** Correspondence:** Y.M, [mei@mail.hzau.edu.cn](mailto:mei@mail.hzau.edu.cn). H.L, [loweliu@whu.edu.cn](mailto:loweliu@whu.edu.cn).

# Supplementary Table 1 Strains used

| Strain | Phylotype | Origin |
| --- | --- | --- |
| *Ralstonia solanacearum* 1 | Ⅰ | BNCC335856 |
| *R. solanacearum*2 | Ⅰ | BNCC335855 |
| *R. solanacearum* 3 | Ⅰ | ATCC BAA-1114 |
| *R. solanacearum* 4 | Ⅰ | Storage in laboratory |
| *R. solanacearum* 5 | Ⅰ | Storage in laboratory  GMI1000 |
| *R. solanacearum* 6 | Ⅰ | GDMCC1.70 |
| *R. solanacearum* 7 | Ⅰ | Preserved at College of Plant Science and Technology, Huazhong Agricultural University |
| *Acinetobacter baumannii* |  | Storage in laboratory |
| *Enterobacter hormaechei subsp* |  |  |
| *Enterobacter cancerogenus* |  |  |
| *Rhizobium rhizogenes* |  | BNCC133856 |
| *Agrobacterium tumefaciens* |  | BNCC180854 |
| *Xanthomonas campestris* |  | BNCC188197 |
| *Ralstonia pickettii* |  | BNCC186282=ATCC27511 |

# Supplementary Table 2 Cas12a targeting sequences and DNA oligos for *in vitro* transcription of crRNAs.

| **Cas12a targeting sequence** | | |
| --- | --- | --- |
| crRNA name | Guide sequence (5′-3′) | PAM (5′-3′) |
| crRNA1(*HrpB*) | GGCCGAUUGCCAGAACGCAUGCU | TTTC |
| crRNA2(*popB*) | ACGCUGGAUCGCGCGCUGCAGGG | TTTC |
| crRNA3(*HrpD*) | GCGGCAGCGCUCGAGCAUUGCGU | TTTG |
| crRNA4(*Egl*) | GCAAGGAUCCGGCGGCCGGUGUC | TTTG |
| **DNA oligos for *in vitro* transcription of crRNAs using T7 polymerase** | | |
| T7-top | taatacgactcactataggg | T7 promoter sequence |
| T7-cR1-oligo | ***AGCATGCGTTCTGGCAATCGGCC***ATCTACACTTAGTAGAAATTAccctatagtgagtcgtatta | Oligonucleotide for *in vitro* transcription of cR1, pairing with T7-top |
| T7-cR2-oligo | ***CCCTGCAGCGCGCGATCCAGCGT***ATCTACACTTAGTAGAAATTAccctatagtgagtcgtatta | Oligonucleotide for *in vitro* transcription of cR2, pairing with T7-top |
| T7-cR3-oligo | ***ACGCAATGCTCGAGCGCTGCCGC***ATCTACACTTAGTAGAAATTAccctatagtgagtcgtatta | Oligonucleotide for *in vitro* transcription of cR3, pairing with T7-top |
| T7-cR4-oligo | ***GACACCGGCCGCCGGATCCTTGC***ATCTACACTTAGTAGAAATTAccctatagtgagtcgtatta | Oligonucleotide for *in vitro* transcription of cR4, pairing with T7-top |

**Supplementary Table 3** Primers used.

| **Primer name** | **sequence (5**′**-3**′**)** |
| --- | --- |
| 1F | CGTTGATGAGGCGCGCAATTT |
| 2F | AAGTTATGGACGGTGGAAGTC |
| AF | ATTACSAGAGCAATCGAAAGATT |
| InF | ATTGCCAAGACGAGAGAAGTA |
| RR | TCGCTTGACCCTATAACGAGTA |
| Hrpb2B3 | GGTGCATCACCAGCTCG |
| Hrpb2F3 | GGTTGGCCAGCAGGGT |
| Hrpb2Bip | GATCGATGGTGGCCAGCCAGCATGCCTCCGACGACGC |
| Hrpb2Fip | CGGCATCCGCTGCTCCAACGCCCATTTCGGCCGATTG |
| HrpB819F | ATGCTGGGAAACATCTACTT |
| HrpB819R | TTCCACCTTGGTCTGCATC |
| 759 | CCGGAATTCGTCGCCGTCAACTCACTTTCC |
| 760 | CCCAAGCTTGTCGCCGTCAGCAATGCGGAATCG |
| FB-reporter | 5′6-FAM-TTTTTATTTTT-3′biotin |
| FQ-reporter | 5′FAM-TTATT-3′BHQ1 |

**Supplementary Figure 1** Multiplex PCR identifies the phylotype of RS in this study.

The two black arrows point to bands that are specific for the *R. solanacearum* phylotype Ⅰ.


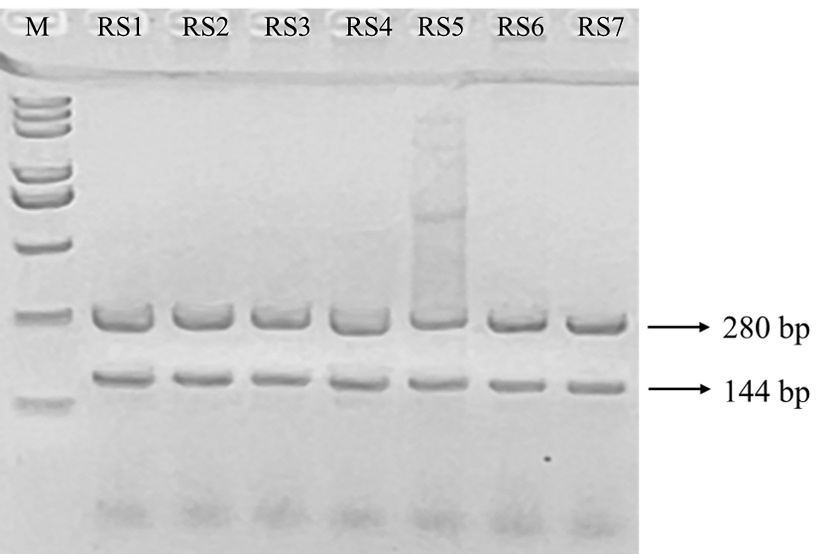


**Supplementary Figure 2** Sequencing results for recombinant plasmid pUC57-*hrpB* (partial).


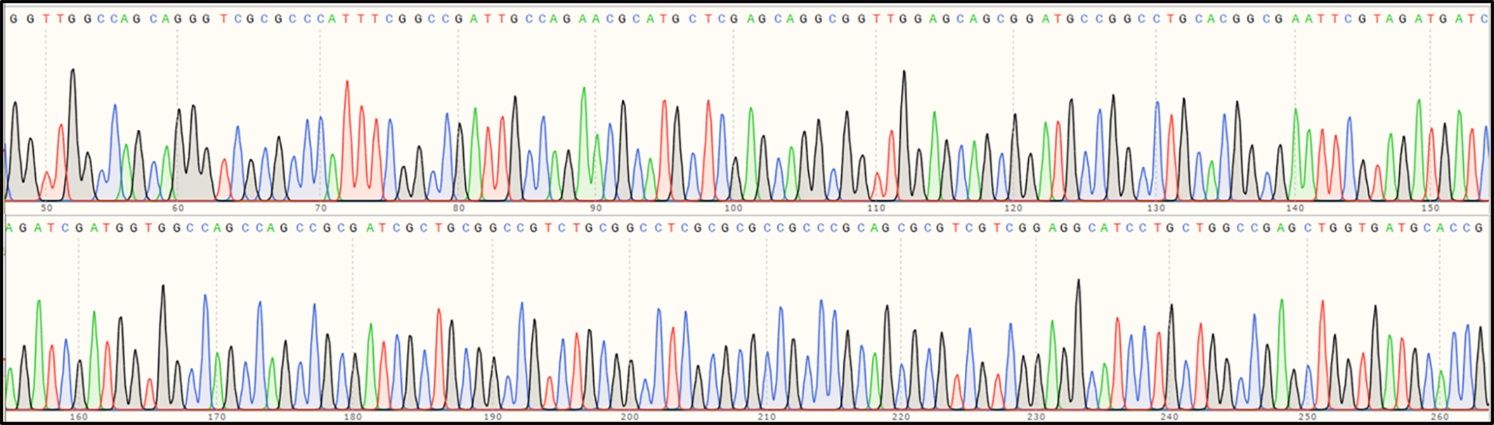


**Supplementary Figure 3 (A)** Optimization of FB-reporter concentration. **(B)** Optimization of PEG amount added to buffer system.

**
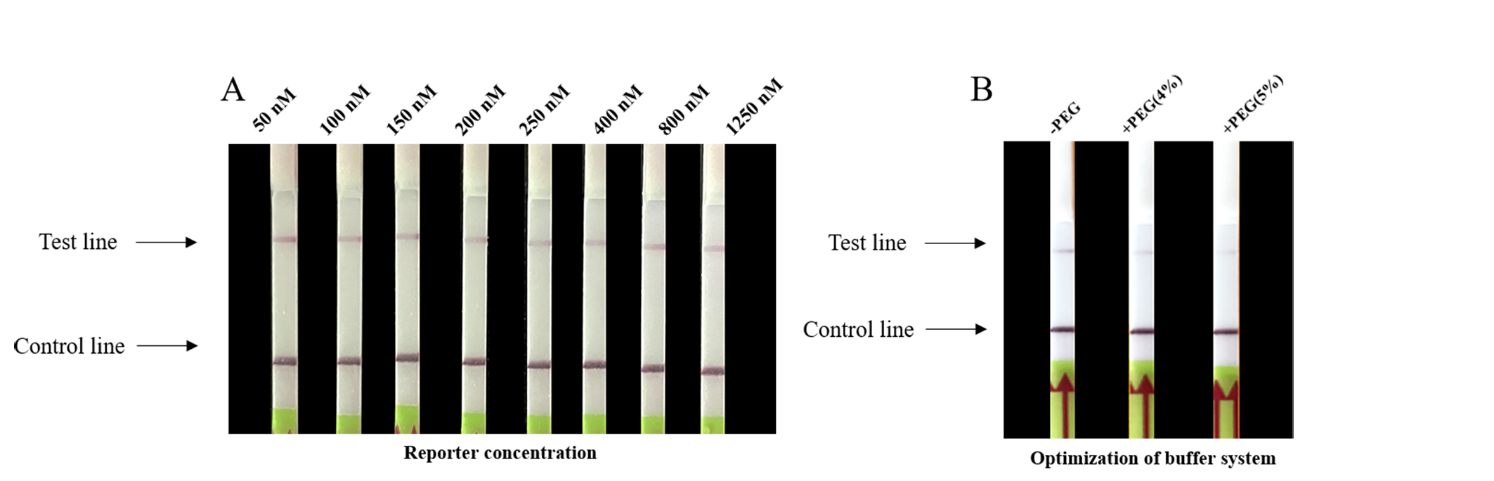
**

**Supplementary Figure 4.** Multiple sequence alignment of *hrpB* genes from various strains and phylotypes of *R. solanacearum*. *hrpb* gene sequences were downloaded from NCBI, and sequence alignment was performed using MEGA7 software program.


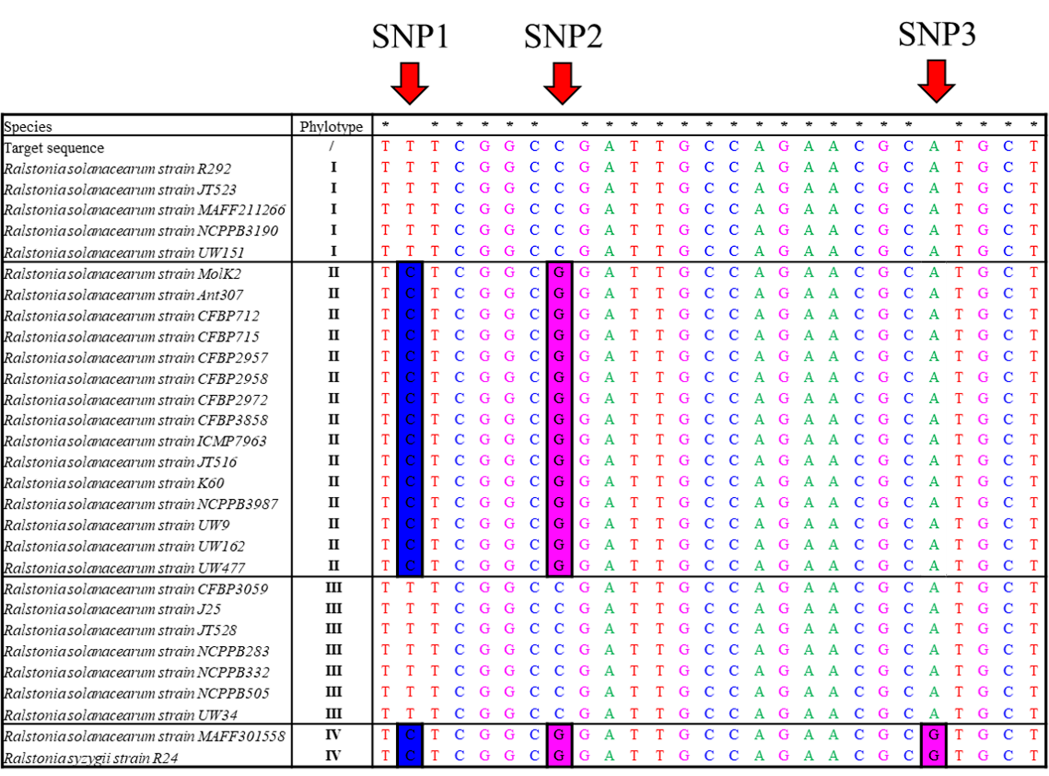

Supplement: Supplementary file 1 [file DataSheet1.docx]
